# Molecular Mechanisms to Control Post-Transplantation Hepatitis B Recurrence

**DOI:** 10.3390/ijms160817494

**Published:** 2015-07-30

**Authors:** Akinobu Takaki, Tetsuya Yasunaka, Takahito Yagi

**Affiliations:** 1 Department of Gastroenterology and Hepatology, Okayama University Graduate School of Medicine, Dentistry and Pharmaceutical Sciences, 2-5-1 Shikata-cho, Kita-ku, Okayama 700-8558, Japan; E-Mail: yasuna-t@cc.okayama-u.ac.jp; 2 Department of Gastroenterological Surgery Transplant and Surgical Oncology, Okayama University Graduate School of Medicine, Dentistry and Pharmaceutical Sciences, 2-5-1 Shikata-cho, Kita-ku, Okayama 700-8558, Japan; E-Mail: liver@cc.okayama-u.ac.jp

**Keywords:** acute hepatitis B, liver cirrhosis, hepatitis B virus vaccine, liver transplantation, hepatitis B immunoglobulin, nucleos(t)ide analogue

## Abstract

Hepatitis B often progresses to decompensated liver cirrhosis requiring orthotopic liver transplantation (OLT). Although newer nucleos(t)ide analogues result in >90% viral and hepatitis activity control, severely decompensated patients still need OLT because of drug-resistant virus, acute exacerbation, or hepatocellular carcinoma. Acute hepatitis B is also an indication for OLT, because it can progress to fatal acute liver failure. After OLT, the hepatitis B recurrence rate is >80% without prevention, while >90% of transplant recipients are clinically controlled with combined hepatitis B immunoglobulin (HBIG) and nucleos(t)ide analogue treatment. However, long-term HBIG administration is associated with several unresolved issues, including limited availability and extremely high cost; therefore, several treatment protocols with low-dose HBIG, combined with nucleos(t)ide analogues, have been investigated. Another approach is to induce self-producing anti-hepatitis B virus (HBV) antibodies using an HBV envelope (HBs) antigen vaccine. Patients who are not HBV carriers, such as those with acutely infected liver failure, are good candidates for vaccination. For chronic HBV carrier liver cirrhosis patients, a successful vaccine response can only be achieved in selected patients, such as those treated with experimentally reduced immunosuppression protocols. The present protocol for post-OLT HBV control and the future prospects of newer treatment strategies are reviewed.

## 1. Introduction

Hepatitis B virus (HBV) infection is one of the main causes of end-stage liver disease requiring orthotopic liver transplantation (OLT). Since HBV is endemic in the eastern hemisphere, the most common indication for OLT in most Asian adults has been HBV-related end-stage liver disease [1]. The post-OLT hepatitis B recurrence rate is >80% without any prevention, while >90% of recurrent infections can be clinically controlled with a combination of hepatitis B immunoglobulin (HBIG) and nucleos(t)ide analogues (NAs) [2]. Recently-available strong- and escape-mutation resistant NAs have been encouraging us to reduce preventive treatment, including frequent life-long administration of HBIG.

The first commercially available NA, lamivudine (LAM), produced a rapid and definite short-term antiviral response, but 15%–20% of the patients who received LAM experienced recurrence of resistant virus each year, and 70% of them did so after five years [3]. Administration of newer NAs such as entecavir (ETV) or tenofovir (TDF) results in resistant virus in fewer than 3% of cases, as they are presently accepted as first-line and long-term treatment, including for liver cirrhosis and post-OLT [4,5,6]. Administration of such newer NAs has enabled the control of HBV-DNA at very low levels, even for liver cirrhosis patients before undergoing OLT. Since HBV-DNA positive status before OLT has been shown to correlate with a high prevalence of recurrence, it is recommended that HBV-DNA-positive patients be strictly followed with relatively higher doses of HBIG [7]. Recently, since newer NAs can induce negative serum HBV-DNA levels for the long term, even for patients with liver cirrhosis, the risk for recurrence post-OLT is decreasing. Some investigators have shown that not using HBIG but only using a newer NA such as ETV, with strict follow-up of HBV-DNA, can result in a very low amount of viral recurrence [8]. So far, a regimen completely without HBIG is not recommended, but with newer NAs, withdrawal of continuous lifelong administration of HBIG might be feasible. Although the prevalence of HBV carriers and genotype distribution differ between western and eastern countries, the prophylaxis methods applied are similar with nearly the same effects (Table 1).

The aims of this article are to review recent advances in molecular mechanisms and the preventive approach for post-OLT HBV, as well as to identify possible ways to protect hepatocytes from HBV infection.

**Table 1:** Representative Post-OLT HBV Prophylaxis with NA and/or HBIG.

Procedures of Post-OLT HBV Prophylaxis	Number of Patients	HBV-DNA Positivity at OLT	HBV-DNA Recurrence	Follow-up (Months)	Reference Number	Year Published	Country
**A. Lamivudine + HBIG**							
HBIG IV 10,000 IU/month	14	7%	0	13	[9]	1998	USA
HBIG IM 400–800 IU/month	141	76%	4%	62 (11–126)	[10]	2007	Australia
**B. Lamivudine + HBIG (on demand)**							
HBIG IV to maintain HBsAb >200 IU/L	21	38%	9.5%	21 (2.4–49.1)	[11]	2001	Germany
HBIG to maintain HBsAb >70 IU/L	11	0%	0	16 (9–22)	[12]	2004	Italy
HBIG IV to maintain HBsAb >10 IU/L	18	61%	0	30 (7–73)	[13]	2007	Japan
Short course (1 month) HBIG	14	0%	7%	18	[14]	2003	Spain
**C. Lamivudine + Adefovir + HBIG**							
LAM + ADV Short course (7 days) HBIG IM 800 IU/day	20	68%	0	57 (27–83)	[15]	2013	Australia
One year HBIG IM 2000 IU/month LAM + ADV or TDF, TDF, ETV	16	4.5%	0	24 (6–40) post HBIG withdrawal	[16]	2012	Greece
**D. Entecavir + HBIG**							
HBIG IM to maintain HBsAb >100 IU/L	63	Average 5.49 × 10^4^ copies/mL	0	41 (33–54)	[17]	2012	China
HBIG dose not specified	61	All cases <172 IU/mL	0	18	[18]	2013	Spain
One year HBIG IV 10,000 U/month	29	52%	3.4%	31	[19]	2013	Korea
**E. Tenofovir + emtricitabine + perioperative HBIG**							
HBIG >6 months to maintain HBsAb >100 IU/L replaced with TDF/EMT	21	56%	0	31 (15–47)	[20]	2012	USA
HBIG >6 months; various protocols	17	88%	0	26 (4–36)	[21]	2013	Netherland
**F. HBIG-free with newer NUCs**							
Entecavir	80	74%	1.2%	26 (5–40)	[7]	2011	China
LAM + ADV (no HBIG when HBV-DNA below 3 log(10)IU/mL)	28	35%	0	22 (10–58)	[15]	2013	Australia
ETV, LAM + ADV, TDF, ETV + TDF (no HBIG when HBV-DNA below 3.3 log(10)IU/mL)	75(Ent42, LAM + ADV19, TFV12, ENT + TFV2)	31%	8%	21 (1–83)	[22]	2013	India

ADV, adefovir dipivoxyl; EMT, emtricitabine; ETV, entecavir; FH, fulminant hepatic failure; HBIG, hepatitis B immunoglobulin; HBsAb, hepatitis B surface antibody; HBV, hepatitis B virus; HBV-DNA, hepatitis B virus DNA; IM, intramuscular; IU, international units; IV, intravenous; LAM, lamivudine; NA, nucleos(t)ide analogue; OLT, orthotopic liver transplantation; TDF, tenofovir.

## 2. Mechanisms of HBV-Related Hepatitis

HBV is an enveloped DNA virus containing a relaxed circular DNA genome enclosed in the envelope, comprising large (L), middle (M), and small (S) proteins [23,24]. The L protein is essential for envelopment and mature virion release, an important function for viral entry [25]. The L, M, and S proteins share the C-terminal S domain, while the L protein includes PreS1 and PreS2 domains, and the M protein includes PreS2 domains in the N-terminus [23]. The C-terminal of PreS1 and the N-terminal of PreS2 are involved in capsid binding, indicative of the infectivity determinant of HBV. A second infectivity determinant is located in the antigenic loop (AGL) of the S domain. The PreS1 domain and the AGL domain are the targets for neutralizing antibodies, while the PreS2 domain targeted antibodies have no neutralizing activities [26,27]. HBIG is composed of AGL-targeted antibodies.

The PreS1 binding sites of hepatocytes have long been sought without success. Using a unique approach of tandem affinity purification combined with mass spectrometry (MS) analysis against a *Tupaia* *belangeri* (tree shrew) hepatocyte proteome database, Yan *et al.* [28] found that the liver bile acid transporter sodium taurocholate cotransporting polypeptide (NTCP) specifically interacts with a key region in the PreS1 domain. This outstanding advancement in HBV virology has yielded several possible approaches to controlling HBV via NTCP down-regulating IL-1β, TNF-α, OSM, or IL-6 administration [29,30,31,32], or NTCP-binding agents such as cyclosporine A [33]. Several drugs such as ouabain, vecuronium, pregnenolone sulfate, bumetanide, irbesartan, and ezetimibe have been shown to inhibit NTCP-mediated transport of bile salts [34,35,36]. Ezetimibe and cyclosporine A have been reported to interfere with HBV entry into hepatocytes, possibly by blocking NTCP function [37,38]. A recent wide screening approach for compounds inhibiting NTCP promoter activity has identified retinoic acid receptor antagonist as a strong candidate for NTCP inhibition [39]. The unveiling of the HBV entry system has the potential to prevent graft liver from HBV infection at OLT.

After envelopment and release of mature virions, HBV is converted into a covalently closed circular (ccc) DNA that persists in the nucleus of infected cells as minichromosomes, which are difficult to eradicate [40]. Once a person is infected, HBV persists in the liver for the rest of a person’s life, even after the patient achieves a clinically cured condition with seroclearance of HBV envelope antigen (HBsAg) and emergence of anti-HBs antibody [41].

In controlling viral replication, immune function has been found to be important, since immunosuppressive treatment for cancer chemotherapy or organ transplantation can induce viral replication even in HBsAg-negative with anti-HBs antibody-positive clinically cured patients and such liver transplanted recipients [42,43].

The HBV, itself, evades the immune system and cell-cycle related system, resulting in a viral-specific and non-specific immune response change and hepatocarcinogenesis [44]. Strong and multi-specific HBV-specific CD4^+^ and CD8^+^ T-cell responses have been shown to correlate with viral and hepatitis control during acute and chronic infection [45,46,47,48]. The interferon-gamma (IFN-γ)-producing anti-viral Type 1 T helper cell (Th1) response against the HBV core has been found to be stronger in patients with resolved infection even several years after infection [49].

The humoral immune response has been acknowledged as useful for understanding the clinical course of acute and chronic hepatitis B [50]. The antibody responds against viral structural antigens such as the core antigen (HBcAg) and the envelope antigen (HBsAg). Anti-HBc IgG antibody (IgG-HBcAb) develops during acute infection and remains positive for the duration of the patient’s life [51]. HBsAg emerges in serum from the acute phase of infection and remains when the patient shows chronic hepatitis while, in patients who experience an acute self-limiting course, HBsAg can be cleared. Anti-HBs antibody is a virus-neutralizing antibody recognized as having lower viral and disease activities. The seroconversion of a person from HBsAg-positive to anti-HBs-antibody-positive is a marker for being able to stop the administration of NAs with success.

## 3. Clinical Characteristics of Post-OLT HBV Recurrence

HBV recurrence has been reported in liver and kidney transplant recipients [52]. A multicenter study in Europe in 1993 identified the risk of post-OLT HBV recurrence [53]. The risk was low in patients with acute liver failure who were intolerant of HBV. However, the recurrence rate in patients with liver cirrhosis, especially with high serum HBV-DNA at OLT, was >80% [53]. Although the immune system in liver transplant recipients is suppressed with steroids and calcineurin inhibitors, recurrent hepatitis B produces high amounts of HBV-DNA and severe lobular hepatitis with a high incidence of fatal liver failure [54]. Todo *et al.* [55] found that, beyond two months after OLT, the mortality, rate of graft failure, and incidence of abnormal liver function tests were significantly higher in the HBV-related group than in the non-HBV related group before the development of antiviral prevention. Co-infection of hepatitis delta virus has been found to result in a better outcome, with a lower frequency of chronic hepatitis recurrence [56,57]. This is explained by the fact that delta virus infection inhibits HBV replication even in immunosuppressed patients [58]. Lerut *et al.* [56] observed that the median time of reappearance of HBV viral markers was 145 days (range 15 to 2615 days) at their center. These investigators reported that three of the 16 HBV re-infected patients showed rapidly progressing fibrosing cholestatic hepatitis and died within one year post-OLT, and two of the 16 patients developed chronic hepatitis with liver failure and died within two years, post-OLT.

At another center, the pathological features of patients with HBV-related liver disease post OLT were reported [9]. Within the first 30 days of OLT, most histological findings were acute cellular rejection, while between 60 and 300 days post-OLT, liver histology showed that acute hepatitis or acute with transition to chronic was the most common diagnosis. Thereafter, a chronic carrier state or chronic active hepatitis and/or cirrhosis were the main features of the biopsy diagnosis. The histological findings of graft failure revealed heterogeneous states such as massive necrosis, cirrhosis, chronic rejection, or hepatic artery thrombosis. The human leukocyte antigen (HLA) matching status with donors did not affect the HBV recurrence rate, indicating that the viral epitope could be recognized via different HLA and T cell receptors.

Since these reports indicated that the time of reappearance of HBV viral markers was similar to the usual acute hepatitis window period, re-infection of the grafted liver could occur early after OLT [10]. Even HBsAg (−) HBc antibody (+) occult HBV carrier blood transfusion could induce hepatitis B transmission with only 1.049 logcopies/mL HBV-DNA required for 50% minimum infectivity [11]. OLT recipients are at risk of perfusion with circulating HBV-DNA and could be easily infected. Since the risk of HBV-DNA positive status before OLT has been shown to correlate with a high prevalence of recurrence, serum is likely the source of infection.

## 4. Past and Present Control of Post-OLT HBV Recurrence with Combination HBIG and NA

Although the HBV recurrence rate without prophylaxis has been very high, present protocols that use NA in combination with long-term HBIG have resulted in >90% control of HBV recurrence [2].

The first trial of long-term HBIG combined with the first-generation NA LAM was conducted in 1998. Monthly HBIG administration with LAM resulted in all patients surviving for one year after OLT without serum HBV-DNA positivity [12]. Subsequent reports also described successful control of HBV recurrence with this combination (Table 1). Since patients with positive HBV-DNA before OLT were more likely to later develop HBV recurrence, maintenance of anti-HBs antibody titers >500 IU/L was recommended in the guideline proposal in 2004 [7]. If HBV-DNA was negative before OLT, the anti-HBs antibody titer could be reduced to 100–150 IU/L with or without LAM. From the standpoint of cost savings, the HBIG dose requirement could be decreased as clinical data accumulated [13,14,59,60]. Currently, HBIG is administered as required only when anti-HBs antibody titers fall below target levels. Some reports indicate that only a short duration of HBIG administration is required and that it can be withdrawn several months after OLT [61]. If HBV-DNA was negative at the time of OLT, HBIG could be withdrawn at several months post OLT. For acute liver failure patients who had been infected with the virus shortly before hepatitis development, HBIG could also be withdrawn. Of course, strict monitoring of HBV-DNA and HBsAg titers should be continued throughout the patient’s life.

Several new NAs, such as adefovir dipivoxil (ADV), entecavir (ETV), telbivudine (LdT), and tenofovir (TDF), have become commercially available [62]. Due to the risk of developing resistance, LAM is no longer recommended as a first-line treatment for hepatitis B. For LAM-resistant virus, add-on adefovir dipivoxil (ADV) or TDF has been proven efficacious for a minimum of two years [63]. For patients who fail the LAM plus ADV combination, ETV plus ADV might be efficacious; Seo *et al.* [64] found that this combination resulted in <3% viral breakthrough. The currently recommended first-line agents are ETV and TDF, which have resulted in a very low emergence of resistance [4,5]. Such newer NAs are very effective when combined with HBIG even during short-duration, post-OLT HBV control [15,16,17,18,19,20,65]. Because of low resistance and the powerful antiviral response evoked by ETV and TDF or a combination of NAs, several institutions have developed successful HBIG-free protocols if the HBV-DNA titer is low enough at the time of OLT [8,21,65]. Since the newer NAs could achieve very low amounts of viral load, even in decompensated cirrhosis, the risk of re-infection at the time of OLT might be reduced. If the recipients have long-lasting HBV-DNA negative status with newer NAs, HBIG could probably be withdrawn after a short duration of administration.

## 5. Mechanisms of Post-OLT HBV Recurrence and Protection

**Figure 1:**
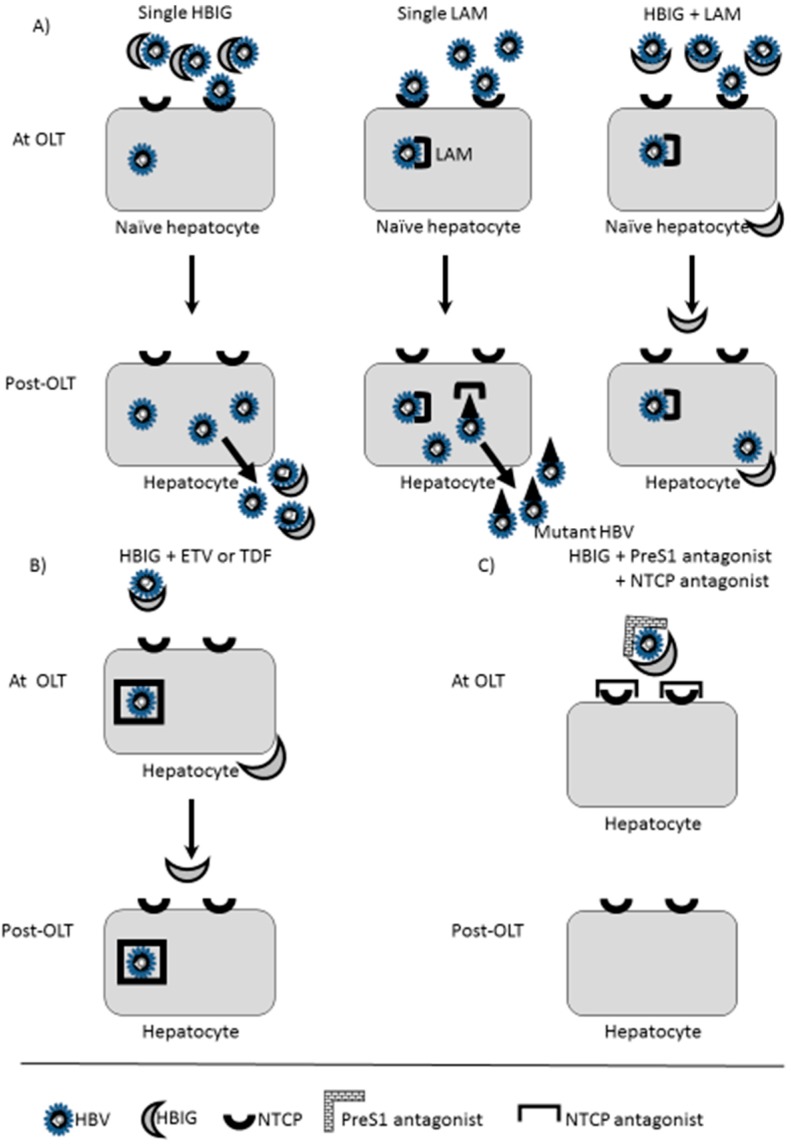
Mechanisms of Hepatitis B Virus (HBV) Prophylaxis after Orthotopic Liver Transplantation (OLT): Past, Present, and Future Perspectives. (**A**) Past: Hepatitis B immunoglobulin (HBIG) single administration or the first-generation nucleoside analogue lamivudine (LAM) resulted in high rates of recurrence. The HBIG and LAM combination controlled HBV recurrence with a relatively high amount of HBIG; (**B**) Present: Since most patients receive newer nuleos(t)ide analogues (NAs) such as entecavir (ETV) or tenofovir (TDF), with very low incidence of viral breakthrough, the HBV viral load before OLT is usually low, and viral control is easier with these drugs. Reductions in the dose of high-cost HBIG have been attempted. HBIG could be used for a short time for pre-OLT negative HBV-DNA patients. The HBV vaccine is good supportive treatment for non-HBV carrier patients and HBV carrier patients with HBV non-tolerated past infected donors or with a lower concentration of calcineurin inhibitors; (**C**) Future: Blocking HBV infection with a combination of HBIG and a PreS1-targeted and NTCP-targeted combination of HBV receptor binding blocking might protect hepatocytes from HBV re-infection.

Although the HBV recurrence rate without prophylaxis has been very high, present protocols that use NA in combination with long-term HBIG have resulted in >90% control. The mechanism of protection against HBV reactivation by the combination of NA and HBIG is not well defined. The cccDNA episome is the transcriptional template for HBV messenger RNA transcripts that encode viral structural and NS proteins and the pregenomic RNA template for reverse transcription and synthesis of the viral genome [22]. NAs inhibit the reverse transcription of pregenomic RNA, resulting in a rapid decrease in serum HBV-DNA, but they cannot eliminate the cccDNA reservoir [66]. HBIG contains high-titer antibodies against the HBsAg L protein AGL domain, which is the major component of the envelope of the HBV virion (Figure 1).

The possible mechanisms through which HBIG prevents HBV transmission are that it: neutralizes circulating virus by immune complex formation; protects naïve hepatocytes against HBV released from extrahepatic sites through blocking the putative HBV receptor; or anti-HBs antibody internalizes into hepatocytes, interacts with HBsAg, and inhibits HBsAg secretion from cells [67]. Protection against HBV infection of naïve hepatocytes might be difficult, since recent studies have demonstrated that intrahepatic HBV-DNA is detectable in >50% of even well-controlled patients post-OLT [22]. The HBV virion released from the infected cells could be blocked with anti-HBs antibody. In an *in vitro* assay, the internalized antibody was seen to induce the accumulation of intracellular viral particles even after the antibody was removed from the cell culture supernatant [68].

## 6. Mechanisms of Post-OLT HBV Prophylaxis Failure

Recent HBV prophylaxis protocols with new NAs have shown very strong effects with very few recurrences. However, long-lasting treatment increases the possibility of NA resistant and HBIG-resistant viruses. HBIG is a blood product obtained from vaccine-based or acute self-limited HBs-Ab positive healthy donors. Acute self-limited HBV hepatitis patients have been shown to have a highly homogeneous viral population, while chronic hepatitis B patients have highly mutant virus, which can be even higher pre or post-OLT [69]. The lower diversity of anti-HBs contained in HBIG would be one reason for HBIG resistance. Even in the case of HBc antibody-positive donor liver received by non-HBV-related cirrhosis patients, hepatitis B recurrence could develop with single HBIG prophylaxis in 25% of cases [70]. These single HBIG prophylaxis-related resistant viruses bore HBV “s” gene mutations such as G145R, G145A, or Q129P. Ultra-deep pyrosequencing (UDPS) analysis of the serum viral DNA of HBV recurrence patients showed that the diversity of HBV quasispecies detected at the time of recurrence was very low in comparison with the diverse quasispecies before OLT [71]. One of the low diversity viruses was, however, able to increase since the virus harbored mutations associated with LAM-resistant “reverse transcription” domain rtT128N which covered the “s” domain sP120T mutation resulting in reduced binding affinity of HBs Ag and HBs Ab. Such a mutant virus showed low proliferating capacity, but it combined into resistant variant rtT128N-rtV173L-rtL180M-rtM204V and showed viral breakthrough. Although such mutants could be treated successfully with ETV, careful follow-up of the patients is needed.

In addition to such escape mutation after HBV prophylaxis, longer administration of new NAs involves several known and unknown problems such as osteomalacia and Fanconi’s syndrome in ADV [72,73]. Since HBIG is a blood-product, infection by unknown organisms or allergic reactions are unavoidable risks. Such patients after HBV-related OLT require life-long general care.

## 7. HBV Vaccine Trial for Post-OLT Patients

The practice of active immunization of post-OLT recipients with HBV vaccine is emerging. For a successful vaccine response, the immune system has important roles. Several genetic polymorphisms of immune system related markers, such as the cytokines interleukin (IL)-1b, IL-2, IL-4, IL-10, and IL-13, and the chemokines CXCR5 and CXCL13 have been reported in non-OLT vaccine effectiveness analysis [74]. In post-OLT patients, most studies reported low response rates, even with doubled concentrations or prolonged injections of vaccines [75,76,77,78,79,80,81,82,83]. Patients who are not HBV carriers (such as adult patients with acute liver failure due to sexual transmission and non-chronic HBV carriers with anti-HBc antibody-positive donor livers) are good candidates for vaccine administration [76,82,84,85,86,87,88]. Patients with acute HBV infection who undergo OLT are often positive for anti-HBs antibody even before OLT, and they have powerful immune responses. Such patients should respond well to vaccination because they have not developed tolerance to HBV, unlike chronic carriers. However, some HBV carriers have responded to vaccination.

### 7.1. HBV Vaccine Trial for Liver Cirrhosis Patients

Since non-carriers respond well to HBV vaccination, even when on prednisolone and calcineurin inhibitor therapy, immune tolerance is expected to play a large role in this process. In non-OLT HBV patients, analysis has revealed that HBsAg-positive newborns had higher regulatory T-cell frequencies and dysfunctional CD8 T cells, which represent an immune-tolerant status [89]. However, another report analyzing the immunological characteristics of HBsAg-positive young carriers and aged patients with active hepatitis showed comparable peripheral T-cell proinflammatory cytokine production capacity and HBV-specific IFN-γ responses [90]. These findings indicate that tolerant carriers can react with HBV antigens and can show active immunity against HBV vaccination, if regulatory T-cell function diminishes. With good responses to newer NAs after OLT, HBV-DNA decreases even in the liver, and this might recover compressed HBV-specific T cells to react with HBV.

Chronic HBV carrier recipients, including patients with positive HBV-DNA at OLT, do not respond well to HBs-antigen-containing vaccine, with response rates being mostly <30% (Table 2) [76,78,79,81,82,83,91]. Tahara *et al.* [86] reported 64.7% positive responses to experimentally minimized immunosuppressant treatment. The immune status of these patients was evaluated by a mixed lymphocyte reaction (MLR) assay in response to anti-donor and anti-third-party allostimulation. The investigators minimized immunosuppression until the donor lymphocytes showed no response as autologous lymphocytes, but third-party lymphocytes showed a positive response. The investigators found that vaccination was successful in patients showing a donor-specific MLR hyporesponse, with a well-maintained response to the third-party stimulus. The vaccine was not successful in patients showing hyporesponse to both donor and the third party. These results provide encouragement that even immune tolerant liver cirrhosis patients can react with HBV vaccines under lower immunosuppressant protocols after OLT.

Monthly to bimonthly repeated vaccine protocols for three years of administration resulted in successful immunization in 40% of patients with post-OLT liver cirrhosis [85]. The donors to good responders were the spouses of recipients and had high anti-HBs antibody titers before donation. The spouses with high-titer anti-HBs antibody were probably infected with HBV by the recipients after marriage, resulting in the strong anti-HBs antibody boost. The immune systems of these donors should not have developed tolerance to the virus [92], and the adoptive immune transfer of the HBV-specific immune response could be achieved [93].

**Table 2:** Representative Post-OLT HBV-Vaccine Trials.

Pre-OLT Disease	Methods	Number of Patients	Definition of Success	Success Rate (%)	Reference Number	Year Published
**Liver Cirrhosis**						
	Novel adjuvant MPL/QS2 vaccine for 0, 4, 16, 18 weeks	16	HBsAb >500 IU/L without HBIG	80	[72]	2007
	Experimental adjuvant vaccine for 0, 1, 2, 6, 12 months	8	HBsAb >500 IU/L 18 months without HBIG	25	[74]	2005
	40 μg for 0, 1, 2, 6, 7, 8 months	18	HBsAb >500 IU/L 12 weeks after last vaccination	0	[70]	2009
	10–20 μg/month with minimal immune suppression	17	HBsAb >100 IU/L without HBIG	64	[78]	2009
	20 μg/month	22	HBsAb >100 IU/L 6 months without HBIG	40	[77]	2012
	20 μg/month	15	HBsAb >100 IU/L 3 months without HBIG	0	[68]	2011
	40 μg 0, 1, 2, 3, months, 20 μg 4, 5, 6 months	50	HBsAb >60 IU/L 3 months without HBIG	24.6	[75]	2013
	40 μg 0, 7, 14, 28 days, 20 μg 2, 3, 4 months	45	HBsAb >60 IU/L 3 months without HBIG	8.8	[75]	2013
	40 μg 0, 1, 6 months	17	HBsAb >10 IU/L without HBIG	82	[69]	2000
	40 μg for 0, 1, 2, 3, 4, 5 months	52	HBsAb >10 IU/L without HBIG	7.7	[73]	2005
**Acute Liver Failure**						
	20 μg/month	5	HBsAb >100 IU/L 6 months without HBIG	100	[77]	2012
	10–20 μg/month with minimal immunosuppression	3	HBsAb >100 IU/L without HBIG	66	[78]	2009
	Experimental adjuvant vaccine for 0, 1, 2, 6, 12 months	2	HBsAb >500 IU/L 18 months without HBIG	100	[74]	2005

HBIG, hepatitis B immunoglobulin; HBsAb, hepatitis B surface antibody.

To successfully transfer immune memory to recipients, the anti-HBs antibody titer of the donors should be high. Luo *et al.* [94] have shown that a high anti-HBs antibody titer (>1000 IU/L) in donors is essential for adoptive transfer. These results suggest that pre-OLT HBV vaccination for candidate living donors might facilitate improved post-OLT vaccine responses in recipients with liver cirrhosis. Although the direct transfer of the donor vaccine-induced anti-HBs antibody stayed only several months [21], these recipients might be good candidates for vaccine administration. Several experimental adjuvant vaccines have also been tried, with success rates up to 44.8% [75,82,95].

The vaccine response depends on immune tolerance to the virus in both recipients and donors. The liver is the largest immune organ in the abdomen; therefore, it plays a critical role in immune responses. Multiple populations of non-hematopoietic liver cells, including sinusoidal endothelial cells, stellate cells located in the sub-endothelial space, and liver parenchymal cells, can function as antigen presenting cells (APCs) [96]. The viral-specific immune competence of the grafted liver might overcome general immune tolerance to the virus in chronic HBV carriers.

In non-OLT chronic hepatitis B patients, those who are HBsAg-negative with anti-HBs antibody positive seroconversion are accepted as candidates for NA discontinuation [97]. However, after OLT, vaccine-induced anti-HBs antibody does not have the same immunological strength as non-OLT self-induced antibody responses. The patients who achieved adequate anti-HBs antibody-positive status after post-OLT vaccine administration with NAs showed 50% HBV breakthrough after cessation of NAs [98]. So far, NA discontinuation is not recommended in post-OLT patient management, even after anti-HBs antibody is achieved with vaccination, since it is difficult even for non-OLT immune competent patients.

### 7.2. HBV Vaccine Trial for HBV-Naïve Recipients Who Received Livers from Anti-HBc Antibody-Positive Donors

Anti-HBc-positive healthy carriers could be candidate donors; this would help address the universal problem of the shortage of donor organs. With regard to the above vaccination protocols, non-HBV-related patients who receive anti-HBc antibody-positive donor livers have favorable results. The post-OLT incidence of *de novo* hepatitis B occurring in anti-HBc antibody-positive donors without prophylaxis is high (33%–100%) [43,99,100]. These HBV-naïve patients are good candidates for the HBV vaccine because 50%–80% tend to respond well [76,82,85,86,87,88]. Pre-OLT vaccination is also possible if patients have sufficient time before undergoing OLT. In countries with universal vaccination programs, the recipients might already have anti-HBs antibody and could be boosted with additional vaccination before OLT, resulting in 78% of prospective recipients having a high titer of anti-HBs antibody (>1000 IU/L) [101]. In pediatric patients, the vaccination responses were observed to be good in recipients with higher anti-HBs titers at the time of OLT and lower tacrolimus levels at the time of vaccination [102].

## 8. Future Perspectives to Control Post-OLT HBV Recurrence

At present, prophylaxis for HBV reactivation is oriented toward viral control with a combination of NAs and HBIG, since more than half of the patients are re-infected. To prevent viral re-infection of HBV-non-infected donor livers, HBIG administration to block HBsAg attachment to hepatocytes is the only method. Many approaches to identify the relevant domain for HBV entry into hepatocytes have revealed that PreS1 is the most probable candidate target for infection. The PreS1 amino acid (aa) positions 2–8 and 18–48 have been shown to decrease binding activities, and the aa 49–78 positions have been shown to increase binding activities [26]. An aa 2–48 consensus sequence derived peptide is named Myrcludex B, and it is being tested in phase 2 trials [23]. A combination of AGL domain-targeted HBIG and PreS1-targeted Myrcludex B might be a possible approach to prevent re-infection.

A more effective vaccine also has the possibility to induce easier and strong HBV prophylaxis. An experimental adjuvant vaccine trial showed induction of a good HBsAb titer response [80]. Recently, several monoclonal antibodies have been shown to induce stronger reactivity to HBsAg, suggesting next-generation vaccine development [103].

Since HBsAg attaches to hepatocyte NTCP (mentioned in section 1), a blocking method could also be directed to NTCP (Figure 1). NTCP participates in the enterohepatic circulation of bile salts and localizes to the sinusoidal/basolateral membrane of hepatocytes. Blocking NTCP results in changes of bile salt transport and might cause hepatocyte dysfunction. Partial inactivation of NTCP to block HBV entry with only a minute decrease of bile transport function might be feasible [38]. 

Combination blocking of HBV infectivity domain PreS1 and AGL and hepatocyte receptor NTCP might hold the possibility of blocking re-infection of HBV and obviating the need for HBV treatment after the short term post OLT.

## 9. Conclusions

Post-OLT hepatitis B recurrence is well controlled with combination use of HBIG and newer NAs, at present. Recent approaches are to reduce the use of HBIG, which is an expensive, blood-related product. The effectiveness of active immunization is dependent upon adaptive immune responses being effective for patients with non-HBV-related disease who have received anti-HBc antibody-positive donor livers, and patients with acute liver failure who are not immune tolerant to HBV. Vaccination is not sufficiently effective for patients with liver cirrhosis; nevertheless, the donor immune memory for HBV and the strength of the immunosuppressant drugs have important roles. Recent advances in research on HBV entry may offer new approaches to protect hepatocytes from HBV re-infection.
